# A microfluidic chip-based co-culture of fibroblast-like synoviocytes with osteoblasts and osteoclasts to test bone erosion and drug evaluation

**DOI:** 10.1098/rsos.180528

**Published:** 2018-09-12

**Authors:** Hui-Peng Ma, Xue Deng, Deng-Yi Chen, Di Zhu, Jin-Ling Tong, Ting Zhao, Jin-Hui Ma, Yan-Qiu Liu

**Affiliations:** 1 College of Laboratory Medicine, Dalian Medical University, Dalian 116044, People's Republic of China; 2 Institute (College) of Integrative Medicine, Dalian Medical University, Dalian 116044, People's Republic of China; 3 People's Liberation Army No. 202 Hospital, Dalian Medical University, Dalian 116044, People's Republic of China

**Keywords:** microfluidic chip, fibroblast-like synoviocytes, bone marrow mesenchymal stem cells, osteoclast, migration, bone erosion

## Abstract

Targeting fibroblast-like synoviocyte (FLS) migration and invasion-mediated bone erosion is a promising clinical strategy for the treatment of rheumatoid arthritis (RA). Drug sensitivity testing is fundamental to this scheme. We designed a microfluidic chip-based, cell co-cultured platform to mimic RA FLS-mediated bone erosion and perform drug-sensitive assay. Human synovium SW982 cells were cultured in the central channel and migrated to flow through matrigel-coated side channels towards cell culture chamber where RANKL-stimulated osteoclastic RAW264.7 and osteogenic medium (OS)-stimulated bone marrow mesenchymal stem cells (BMSC) were cultured in the microfluidic chip device, mimicking FLS migration and invasion-mediated bone erosion in RA. These SW982 cells showed different migration potentials to osteoclasts and BMSC. The migration of SW982 cells with high expression of cadherin-11 was more potent when SW982 cells were connected with the co-culture of RAW264.7 and BMSC. Simultaneously, in the co-cultured chamber, tartrate-resistant acid phosphatase (TRAP) activity of RANKL-stimulated RAW264.7 cells was enhanced, but alkaline phosphatase (ALP) activity was decreased in comparison with mono-cultured chamber. Furthermore, it was confirmed that celastrol, a positive drug for the treatment of RA, inhibited SW982 cell migration as well as TRAP activity in the cell-cultured microfluidic chips. Thus, the migration and invasion to bone-related cells was reconstituted on the microfluidic model. It may provide an effective anti-RA drug screen model for targeting FLS migration-mediated bone erosion.

## 1. Introduction

Rheumatoid arthritis (RA) is a chronic systemic auto-immune disease, characterized by joint synovitis. Abnormal proliferation and migration of fibroblast-like synoviocytes (FLS) play key roles in RA pathogenesis [1,2]. RA FLS activation and migration increase activation of proinflammatory pathways [3,4] and secretion of matrix-destructive enzymes, such as MMPs, thereafter promotes bone erosion [5]. Although bone erosion is a secondary factor in RA, bone erosions by FLS-mediated synovitis has now become a central element in the diagnosis, treatment and monitoring of RA [6]. Bone erosion represents localized bone loss resulting from an imbalance in which bone resorption by osteoclasts is favoured over bone formation by osteoblasts. Understanding the mechanisms that define the formation of bone erosions requires insight into the interaction of FLS with osteoclasts and osteoblasts [7]. However, most assays target only FLS or bone cells, which is extremely disparate from RA conditions *in vivo*. Furthermore, even though some assays can select efficacious drugs, most fail to target both FLS-mediated synovitis and bone erosion. Therefore, it is vital to develop a straightforward and reliable platform to assay the interaction of FLS with bone cells as well as drug sensitivity to guide the treatment of RA.

Microfluidic chip technology has been well accepted by the biological and medical research communities [8–10] as a powerful tool for reconstructing microenvironments at tissue, cellular and molecular level [11,12]. Microfluidic technology permits the accommodation and control of micro- to pico-litre amounts on a device measuring a few square centimetres or even smaller. Consequently, it miniaturizes basic conventional biological or chemical laboratory operations, such as sample preparation, reaction, separation and assay [13]. Compared to conventional static approaches, microfluidic-based cell cultures are able to continuously observe cell migration and cell–cell communication [14] in a defined microenvironment such as bone erosion microenvironment [15,16]. This could overcome the limitation of the conventional up-down chamber culture, in which is difficult to reconstruct such multicellularity and spatio-temporal complexity [17–19]. In addition, it is hard to achieve high-throughput screening with the strategies currently used due to their high consumption of reagents and power, long reaction time and tedious operation process.

In the present study, we developed a microfluidic co-culture drug evaluation platform on which FLS was indirectly co-cultured with osteoclasts and bone marrow mesenchymal stem cells (BMSC), the precursor of osteoblasts. The resulting model represents a functional cell co-culture mimicking FLS migration and bone erosion in which FLS migrates towards the mixture of BMSC and osteoclasts through collagen-incorporated microchannels. Then we used this model to evaluate the ability of anti-RA agent of celastrol to inhibit FLS-mediated bone erosion.

## 2. Material and methods

### 2.1. Fabrication of the microfluidic array

**Figure 1.**
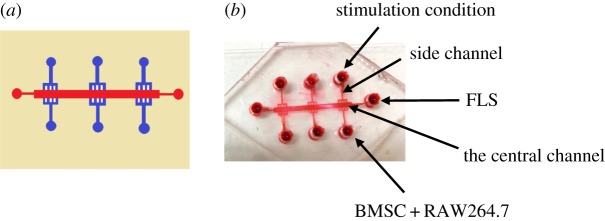
Reconstitution of the microfluidic model. (*a*) The design of the microfluidic model. This model is composed of two layers: one layer of glass substrate, another layer of a PDMS membrane. There are six parallel branched microchannels that are joined at the cell reservoir at one end and joined at the centre channel at the other end on the top PDMS layer. (*b*) Photos of the established microfluidic model.

The schematic diagram of the microfluidic devices is shown in figure 1. It was designed to contain three co-culture units in a single device. Each unit contains one central channel for FLS culture and two side channels, which lie at both sides of the central channel and are used for supplementation with growth medium. The height of the central channel was 180 µm, while the height of other parts on the device was 65 µm. The height of the side channel was designed to be lower than that of the central channel so that the two different types of cells can be introduced into the chambers sequentially. The master was prepared by double coating SU8-3035 (Microchem, Newton, MA, USA) onto a glass wafer and patterned by photolithography. Then, polydimethylsiloxane (PDMS) precursor and curing agent (10 : 1 w/w) were mixed thoroughly, degassed under vacuum and poured onto the master. The assembly was cured in an oven for 2 h at 80°C. After cooling, the PDMS slab was gently peeled from the master and trimmed to size. The PDMS layer and clean glass substrate could be irreversibly sealed by plasma treatment for 45 s. The bonded devices were quickly placed under the UV light for 30 min and cell suspension can be introduced subsequently.

### 2.2. Gelation of matrigel in microchannel

Matrigel was pre-coated in the microchannels via the cell reservoir within 25 min after the microchip was prepared. The injection volume was 0.4 µl. The microchips were placed on the culture plates and incubated at 37°C for 30 min. The microchips were washed with PBS prior to culture of cells.

### 2.3. Culture of mouse bone marrow mesenchymal stem cells

BMSC were isolated according to a previously published protocol with some modification [20,21]. Briefly, BMSC were isolated from bone marrow, aspirated from 8-week-old BALB/c mice. BMSC were collected using gradient centrifugation of mesenchymal stem cell-specific gradient solutions (Tianjin Haoyang Biological manufacture Co., Ltd, China). A layer of PBS-buffered bone marrow cell fraction was placed on the top of gradient solution and centrifuged at 340*g* for 20 min. The cell fraction was collected and washed with PBS. The cell samples were resuspended in Minimum Essential Medium Alpha Medium (α-MEM, Gibco, Paisley, UK), supplemented with 10% fetal calf serum (FCS), 100 U ml^−1^ penicillin and 100 µg ml^−1^ streptomycin, and maintained at 37°C with 5% CO_2_ in a humidified atmosphere. On day 3, the cell suspension was decanted and it was replaced with fresh complete medium. BMSC were further separated from haematopoietic cells by their differential adhesion to tissue culture plastic and their prolonged proliferation potential. Upon 6–7 days culture, 90% of cell confluence was reached. These cell samples were employed with the experiment.

### 2.4. Culture of pre-osteoclastic RAW264.7 cells and SW982 cells

Mouse pre-osteoclastic RAW264.7 cells and human synovial sarcoma SW982 cells were purchased from the Type Culture Collection of Chinese Academy of Sciences (Shanghai, China). The cells were cultured in DMEM (Gibco, Grand Island, NY, USA) supplemented with 10% FCS, 0.03% l-glutamine (Gibco), penicillin (100 U ml^−1^) and streptomycin (100 µg ml^−1^), and maintained at 37°C with 5% CO_2_ in a humidified atmosphere.

### 2.5. Cell co-culture in the microfluidic device

FLS (1 × 10^5^ per ml) were cultured in the centre channel after the collagen is solidified. RAW264.7 cells (1 × 10^4^ per ml) and BMSC (1 × 10^4^ per ml) were added to the side chamber, separately or together. Cells were adapted to DMEM for 3 days before being cultured in the microfluidic device and maintained at 37°C with 5% CO_2_ in a humidified atmosphere.

For *in vitro* osteoblast differentiation, BMSC were pre-cultured with osteogenic medium (100 nM dexamethasone, 1 mM β-glycerophosphate and 5 µM L-ascorbic acid 2-phosphate) for 5 days. Culture medium was changed every third day. After 9 days, alkaline phosphatase (ALP) staining (Sigma) was performed according to the manufacturer's instruction.

For osteoclast differentiation, cells were plated in DMEM with 50 ng ml^−1^ recombinant RANKL for 4 days.

### 2.6. Migration assay

The migration distance was photographed at the indicated time points using a TE2000-U microscope (Nikon Instruments, Melville, NY, USA). The rate of migration was calculated by measuring the distance from the central channel to the side channel as follows:

$$\text{Rate of migration in \%}=\frac{\text{distance moved}\;(\text{migrating cell front})}{\text{side channel distance}}\times 100\text{\%}.$$

### 2.7. Immunofluorescence staining

For immunfluorescence of FLS, the cultures were fixed in 4% paraformaldehyde (PFA) for 20 min and then permeabilized with 0.25% Triton-X 100 in PBS. Cells were then blocked by incubation with a blocking solution containing 10% goat serum before primary antibody incubation at 4°C for 24 h. The cells were incubated with TBST overnight with gentle rocking at 4°C and then further incubated with secondary antibodies (Cell Signaling Technology). Primary antibodies used were rat cadherin (1 : 100, Cell Signaling Technology); to avoid fluorescence quenching, a drop of anti-fade gold reagent with DAPI (Cell Signaling Technology) was added on top of the fixed/stained cultures before imaging. The fluorescence images were captured by an Olympus inverted fluorescent microscope.

### 2.8. ALP staining

ALP staining measurement was performed by using an ALP staining kit according to the manufacturer's instruction (Sigma). Briefly, cells were fixed with 60% citrate-buffered acetone for 30 s. Then the cells were stained for ALP with 0.1 M acetate solution (pH 9.0) containing 6.76 mM sodium tartrate, 0.12 mg ml^−1^ naphthol AS-MX phosphate and 0.07 mg ml^−1^ of Fast Garnet GBC solution, as described in the manufacturer's instruction (Sigma).

### 2.9. TRAP staining

For tartrate-resistant acid phosphatase (TRAP) staining, cells were fixed with 60% citrate-buffered acetone for 30 s. Then the cells were stained for TRAP with 0.1 M acetate solution (pH 5.0) containing 6.76 mM sodium tartrate, 0.12 mg ml^−1^ naphthol AS-MX phosphate and 0.07 mg ml^−1^ of Fast Garnet GBC solution, as described in the manufacturer's instruction (Sigma). Photomicrographs were obtained using an Olympus microscope at 200× magnification.

### 2.10. Double staining of ALP and TRAP

Double staining was performed, as we described previously [22]. Cells were fixed with 60% citrate-buffered acetone for 30 s. Then the cells were stained for ALP with an ALP staining kit. After the reaction solution was removed and discarded, the cells were washed with deionized water. The cells were further stained for TRAP with 0.1 M acetate solution (pH 5.0) containing 6.76 mM sodium tartrate, 0.12 mg naphthol AS-MX phosphate ml^−1^ and 0.07 mg of Fast Garnet GBC solution ml^−1^, as described in the manufacturer's instruction (Sigma). Photomicrographs were obtained at 200× magnification.

### 2.11. Statistical analysis

Differences between experimental groups were evaluated by one-way analysis of variance (ANOVA) using SPSS 17.0 software. Differences with a *p*-value < 0.05 were considered statistically significant. All experimental data are presented as the mean ± s.e.m. with values from more than three experiments.

## 3. Results

### 3.1. Generation of RA bone erosion model using FLS, BMSC and RAW264.7 cells

The microfluidic model was composed of two layers: one layer of glass substrate and another layer of PDMS membrane, which is widely used in microfluidic platforms for biological research because of its good biocompatibility and gas permeability. The PDMS layer contained six parallel microchannels (65 µm height and 400 µm width) that are joined at the cell reservoir at one end and joined at the centre channel at the other end. The established microfluidic model is shown in figure 1.

### 3.2. Effect of FLS migration in BMSC and osteoclastic RAW264.7 cell co-cultured microfluidic array

**Figure 2.**
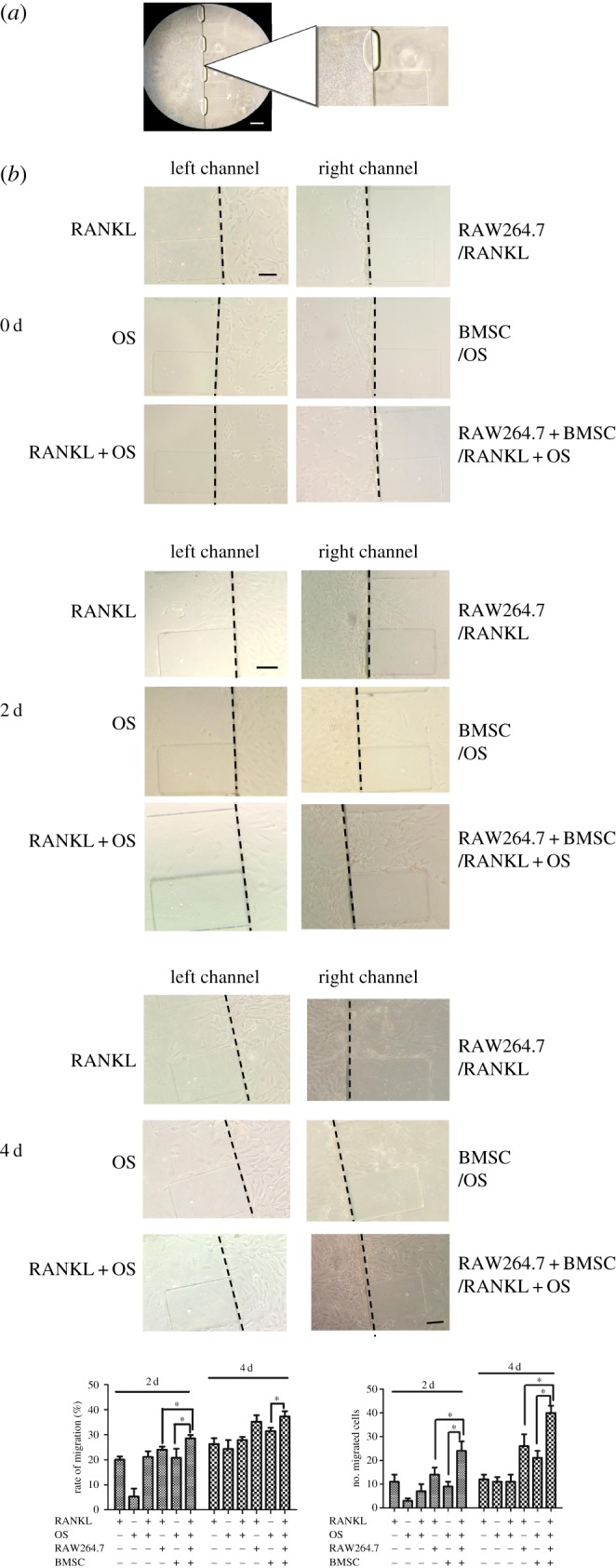
Migration of synovial SW982 cells co-cultured with osteoclastic RAW264.7 cells and BMSC in microfluidic chip. (*a*) Gelation of matrigel in side microchannel. Matrigel was added to the side microchannel and incubated for 30 min to gelation prior to culture of SW982 cells. Cells were imaged at 100×. Scale bar is 100 µm. (*b*) Migration activity of SW982 cells in different stimulus conditions in the microfluidic chip. SW982 cells were cultured in the centre channel, while RANKL-stimulated RAW264.7 cells and OS-stimulated BMSC were cultured in the chamber. The migrated number and migration rate of SW982 were determined after incubation for different time. Cells were imaged at 200×. Scale bar is 50 µm. Data are expressed as mean±s.e.m. of three independent experiments. *p < 0.05.

To mimic the organism barrier between synovial organism and bones *in vivo*, matrigel, a substitution of extracellular matrix, is pre-coated in the microchannels via the cell reservoir at first (figure 2*a*). The bubbles in microfluidic chip, shown in figure 2*a*, were formed at sealing the PDMS layer with clean glass substrate. It did not affect the migration assay. FLS were loaded into the centre channel via the individual inlet. FLS were attached to the centre channel and they formed a monolayer. At the cell reservoir, pre-osteoclastic RAW264.7 and BMSC were cultured to establish a synovium and bone organ microenvironment. RANKL and OS were added to stimulate osteoclast differentiation and osteoblast differentiation, respectively. Upon 4 days stimulation, FLS migrated towards different co-cultured conditions at different degree. Upon 2 days culture, FLS started to migrate. Compared with RANKL or OS stimulation group, the migrated number of FLS and the migrated extend increased when FLS were co-cultured with BMSC, osteoclastic RAW264.7 cells or BMSC plus RAW264.7 cells. The migrated number of FLS was the most potent when the cell reservoir was cultured with BMSC and osteoclastic RAW264.7 cells. After the cells were treated for 4 days on the microchip, the migrated number was further increased. Similarly, in BMSC and RAW264.7 co-culture group, the increased number and distance of migrated FLS were more significant than that in BMSC or RAW264.7 group (figure 2*b*).

### 3.3. Cadherin-11 expression was altered in SW982 cells co-cultured with BMSC and RAW264.7 in microfluidic array

**Figure 3.**
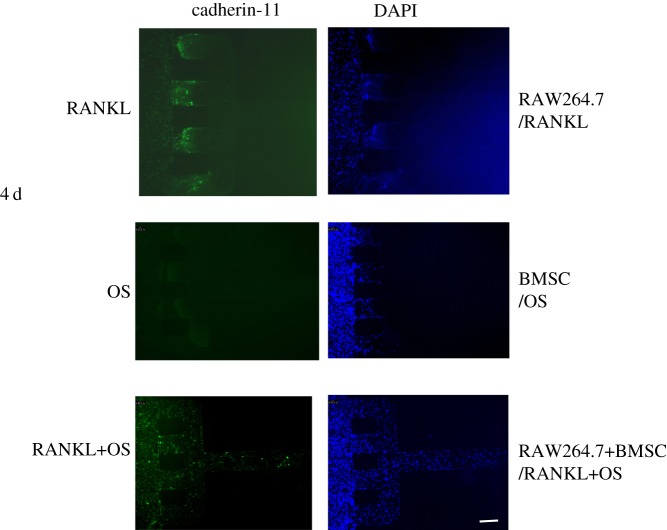
Expression of cadherin-11 on SW982 cells. SW982 cells were co-cultured with RAW264.7 cells and BMSC on the microfluidic and incubated for 4 days. Immunofluorescent staining was performed after stimulation with RANKL and OS for 4 days. The fluorescence images were captured by an Olympus inverted fluorescent microscope. Cells were imaged at 100×. Scale bar is 100 µm.

Cadherin-11 is considered a mesenchymal cadherin. Expression of cadherin-11 correlates with tissue outgrowth and tissue extension. Recent studies demonstrated aberrant expression of cadherin-11 in synovial pathology that was associated with an increased invasive phenotype and RA progression. To investigate the expression of cadherin-11 by FLS in the microfluidic chip, immunofluorescence staining was performed. When FLS were co-cultured with RAW264.7 cells and/or BMSC in the microfluidic chip, cadherin-11 expression levels were different. Compared with the group of BMSC, co-culture with RAW264.7 cells resulted in an increase in the expression level of cadherin-11. Especially, migrated FLS showed high levels of cadherin-11 expression. When FLS were connected with RAW264.7 and BMSC, more migrated FLS expressed cadherin-11 (figure 3).

### 3.4. Alteration of ALP and TRAP activity after co-culture of BMSC, RAW264.7 and FLS in microfluidic chip device

**Figure 4.**
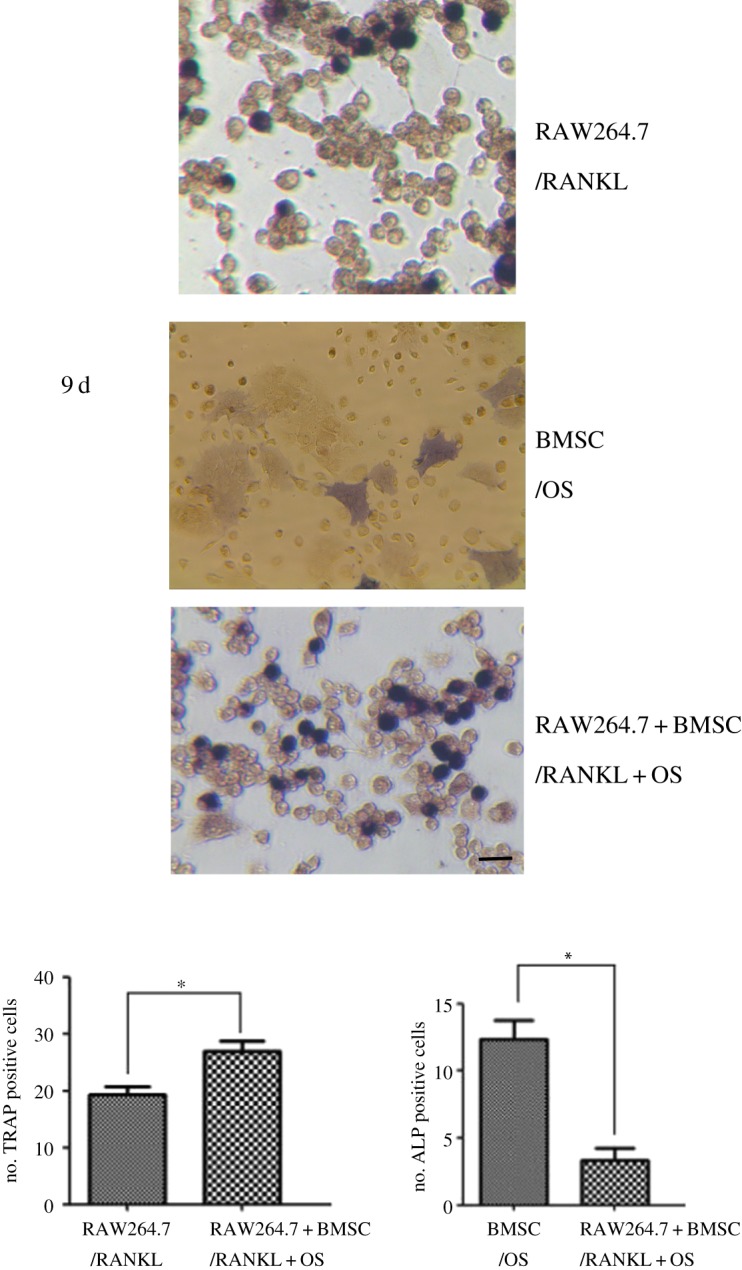
Altered activity of TRAP and ALP in osteoclastic RAW264.7 cells and BMSC in the co-cultured microchip devices. The activities of TRAP and ALP at different stimulation case, including addition of RANKL or OS monocultures or co-cultures, are presented on day 9 of the cultures. The representative photomicrographs were made on cultures fixed on day 14. The ALP is stained with naphthol AS-MX alkaline solution containing a diazonium salt, while TRAP is labelled by naphthol AS-BI in conjunction with a diazonium salt. The number of ALP and TRAP positive cells was measured. Data are expressed as mean ± s.e.m. of three independent experiments. *p < 0.05. Cells were imaged at 200×. Scale bar is 50 µm.

To investigate the interacted influence through co-culture of BMSC, RAW264.7 and FLS in microfluidic chip device, activities of ALP, a marker for osteoblast differentiation and TRAP, a marker for osteoclast differentiation were assayed. When RAW264.7 cells co-cultured with FLS were stimulated with RANKL for 4 days, TRAP staining-positive cells were observed. Similarly, when BMSC co-cultured with FLS were stimulated with OS for 9 days, ALP staining-positive cells were observed. By contrast, the number of TRAP staining-positive RAW264.7 cells increased, but the number of ALP staining-positive cells decreased after co-culture of BMSC, RAW264.7 and FLS (figure 4), indicating that FLS activation and migration enhanced osteoclastic activity but inhibited osteoblastic differentiation.

### 3.5. Activity of celastrol was identified in the microfluidic chip device

**Figure 5.**
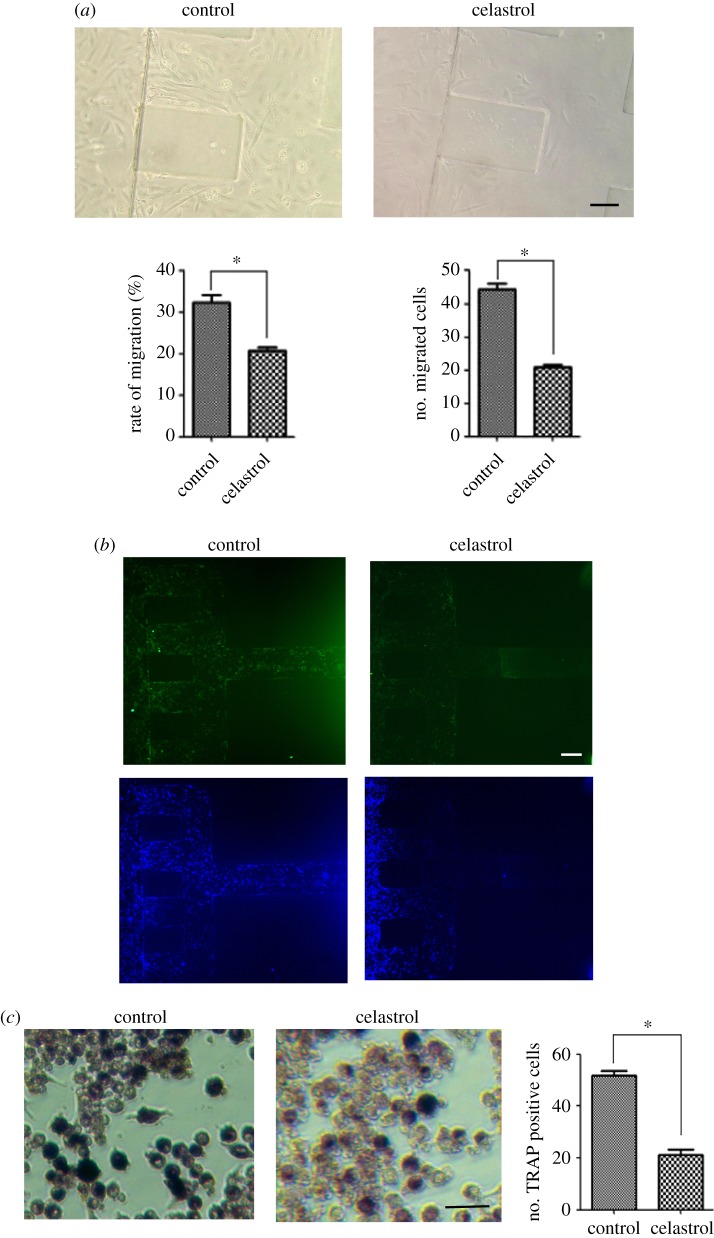
Effect of celastrol on FLS migration, cadherin-11 expression, TRAP and ALP activity in the co-cultured microchip devices. The co-cultured cells were exposed to celastrol and fixed on day 4. (*a*) Migrated FLS were observed by microscopy. The migrated number and migration rate of SW982 were measured. Cells were imaged at 200×. Scale bar is 50 µm. Data are expressed as mean ± s.e.m. of three independent experiments. *p < 0.05. (*b*) Cadherin-11 expression was assayed by immunofluorescent staining. Cells were imaged at 100×. Scale bar is 100 µm. (*c*) TRAP activity was stained by using TRAP kits. The number of TRAP positive cells was measured. Data are expressed as mean±s.e.m. of three independent experiments. *p < 0.05. Cells were imaged at 200×. Scale bar is 50 µm.

Celastrol (tripterine) is a chemical compound isolated from the root extracts of *Tripterygium wilfordii* (thunder god vine) and *Celastrus regelii*. Celastrol has been confirmed to be effective for treating RA. Celastrol is reported to inhibit FLS activation and attenuate bone erosion in collagen-induced arthritis mice and inhibits osteoclast differentiation and function *in vitro* and *in vivo*. Therefore, we used celastrol as a positive drug to evaluate the microfluidic chip model for FLS-mediated bone erosion. Different doses of celastrol in FLS migration were screened. We found that 500 ng ml^−1^ celastrol obviously inhibited FLS migration. This result was similar to previous studies [23,24]. When 500 ng ml^−1^ celastrol was added to the co-culture in the microfluidic chip device and incubated for 4 days, the number of FLS migration decreased when compared with untreated control (figure 5*a*). The expression of cadherin-11 in both migrated and non-migrated FLS was reduced after the co-culture was treated with celastrol (figure 5*b*). Also, the number of TRAP staining-positive cells was decreased in the presence of 500 ng ml^−1^ celastrol (figure 5*c*).

## 4. Discussion

The advantage of biomimetic microfluidic models is to reproduce complex and integrated physiological and pathological events, which can be used as important alternatives to animal models in basic biological research and drug development. In this study, we developed an efficient, accurate and high-throughput microfluidic chip-based drug sensitivity test platform. With this device, the synovial SW982 cell line was co-cultured indirectly with osteoclastic RAW264.7 cells and BMSC with the continuous supplementation of medium, mimicking the actual condition of RA FLS migration and invasion-mediated bone erosion. Previously, FLS migration was determined by using wound scratch assays [2,25]. In this device, the real-time process of FLS migration and invasion can be observed through the microchannels. The infiltration of FLS may be caused by FLS migration or proliferation. The mobility of FLS was different under different conditions of cell co-culture in the microfluidic device. FLS and bone cells including osteoblasts and osteoclasts can interact with each other to influence their activities and function, resulting in bone erosion in RA [26]. By adding matrigel to the microchannels, FLS in synovium and bone-related BMSC and osteoclasts were isolated and cultured in different vessels and retained many characteristics of the original organs, such as cell migration and functional activities. However, cytokines and chemokines could influence intercellular interaction to affect cell migration and functional activities [27–29]. Overall, cells with synovium and bone organ properties and microvessels with matrigel were integrated in the microfluidic model, and the migrated potential of FLS towards bone cells could be assessed. The synovial cell line SW982, tested here, showed significant migration to BMSC and osteoclastic RAW264.7 cells. These results were consistent with clinical observations. Together, the results demonstrated that the multiple cell culture model provided a novel platform for the study of FLS migration and invasion-mediated bone erosion in RA.

Through connection of FLS with RAW264.7 or BMSC, functionally reciprocal impact occurred. SW982 cell migration accelerated when the cells were connected with differentiated RAW264.7 cells. This result was consistent with previous studies that FLS produced RANKL by IL-17 and thereafter promoted osteoclastogenesis [27]. RANKL-stimulated RAW264.7 cells were differentiated towards mature osteoclasts, in which process a variety of cytokines and chemokines were produced [28], therefore promoting SW982 cell migration. By contrast, SW982 cell migration rate was low when the cells were connected with BMSC, suggesting that BMSC might produce less chemokines than RAW264.7 cells did. Although synovial cells were implicated to have the ability to induce bone mineralization through production of IL-26 or miRNA, [29] in the co-culture of FLS, RAW264.7 and BMSC, the number of ALP staining-positive cells decreased, but the number of TRAP staining-positive cells increased. Our previous study showed that BMSC and RAW264.7 cell co-culture increased the activity of ALP, but did not affect TRAP activity [22]. These results combined with current results suggested that FLS co-cultured with RAW264.7 and BMSC influenced the activity of BMSC and RAW264.7 cells. The interaction mechanism among these three types of cells needs to be further studied.

Cadherin-11 plays an important role in FLS migration and invasion [30]. It is a type II cadherin predominantly expressed by FLS residing in the synovium. Recent research showed that cadherin-11 could regulate inflammation mediated by FLS [31] and promote migration of FLS and erosion of cartilages and bones [32]. Cadherin-11 promotes the secretion of IL-6, a critical inflammatory factor that promotes FLS activation and migration [33,34]. Cadherin-11 was strongly expressed when FLS were connected with RAW264.7 cells. The expression was more potent after FLS were connected with BMSC and RAW264.7 cells. The results were similar to previous studies that expression of cadherin-11 enabled prostate cancer (PCa) cells to intercalate into osteoblasts and increased the migration and invasion of PCa cells [32]. In migrated FLS, cadherin-11 seemed to have higher expression level, suggesting a correlation of caherin-11 with FLS migration and invasion [35]. The expression level of cadherin-11 was relatively low in the presence of BMSC. Also, when umbilical cord-derived mesenchymal stem cells (UCMSC) were co-cultured with FLS, cadherin-11 expression was decreased in comparison with RA FLS monoculture [36].

To investigate whether this microfluidic model could be used to effectively screen drugs for their potential to inhibit FLS-mediated bone erosion, the ability of celastrol to inhibit FLS migration was determined using the microfluidic chip. Celastrol was able to inhibit SW982 cell migration even SW982 cells were co-cultured with RAW264.7 cells and BMSC in the microfluidic device. Previous studies showed that celastrol inhibited FLS through suppressing HIF-1*α*/CXCR4 signalling pathway [37] or TLR4/NF-κB-mediated matrix metalloproteinase-9 expression [38]. Celastrol was also reported to attenuate bone erosion in collagen-induced arthritis mice and inhibits osteoclast differentiation and function in RANKL-induced RAW264.7 [39]. In the microfluidic device, celastrol inhibited SW982 migration and simultaneously suppressed TRAP activity in RAW264.7 cells, suggesting a cellular mechanism of celastrol by which celastrol exerted an effective treatment in RA rats through preventing bone loss and bone microarchitecture degradation [40]. We also found that celastrol did not enhance ALP activity of BMSC. The result was similar to previous report that celastrol decreased the number of osteoblasts in arthritic joints of RA rats [41]. Therefore, our results of celastrol in the impact on FLS migration, osteoclastic activity and osteoblast activity in the cell co-cultured microchips were consistent with the result of celastrol *in vivo*, suggesting a more real mimicking of RA *in vitro*.

## 5. Conclusion

In summary, we have developed a microfluidic cell co-cultured model to reproduce FLS migration-mediated bone erosion in RA. Comparison of different co-culture conditions demonstrated that functionally reciprocal impact was more potent when FLS were co-cultured with RANKL-stimulated RAW264.7 cells and OS-stimulated BMSC. The activity of celastrol was confirmed to synchronously inhibit SW982 migration and TRAP activity in the microfluidic model. This study provides an effective model *in vitro* to predict the migration of FLS towards bone-related cells and to rapidly screen possible anti-RA drugs.

## Data accessibility

This article does not contain any additional data.
